# How Sociotechnical Realignment and Sentiments Concerning Remote Work are Related – Insights from the COVID-19 Pandemic

**DOI:** 10.1007/s12599-023-00798-8

**Published:** 2023-03-24

**Authors:** Vanessa Kohn, Muriel Frank, Roland Holten

**Affiliations:** Chair of Information Systems Engineering, Goethe University Frankfurt, Theodor-W.-Adorno-Platz 4, 60323 Frankfurt am Main, Germany

**Keywords:** Sociotechnical systems theory, Q methodology, Sentiment analysis, Resilience, Remote work, Work from home

## Abstract

The COVID-19 pandemic forced sociotechnical systems (STS) to highly integrate remote work. Large-scale analyses show that the positivity of tweets about work from home decreased until COVID-19 was declared a pandemic by the WHO and re-increased in the weeks that followed. Nevertheless, it is unclear if this reaction is due to personal and organizational developments or if it mirrors the realignment of entire STS. The present study uses Q methodology to identify differences in how STS realign to the externally enforced integration of remote work. Only STS that reach a state of high alignment to remote work conditions by successfully shifting communication and procedures to digital spheres can be considered resilient. The results show that employees describe their personal experiences with remote work as more positive the higher their level of sociotechnical realignment. Furthermore, personal digital resilience is correlated to successful STS realignment as well. The results confirm the importance of realigning not only the technical and social components of STS but above all their sociotechnical interaction. Negative sentiments relate in particular to the low realization of humanistic objectives in STS.

**Supplementary Information:**

The online version contains supplementary material available at 10.1007/s12599-023-00798-8.

## Introduction

**Fig. 1:**
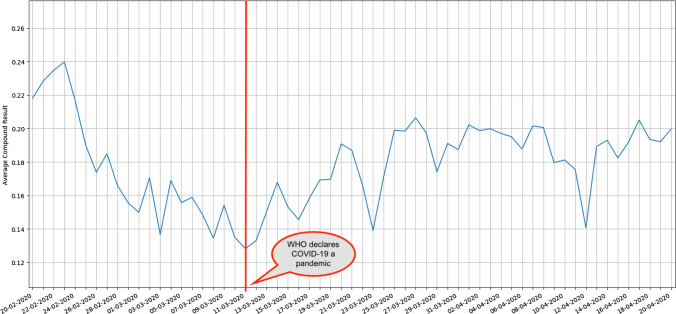
Average daily compound scores (20 February 2020–20 April 2020) (Kohn 2020)

The ongoing COVID-19 pandemic has catapulted a majority of people into remote work. The Federal Statistical Office (2020) indicates that back in 2019 only 13% of those employed worked at least partially from home. However, during the COVID-19 pandemic, this number increased to 60% (Statista 2020). Many struggled with this transition (Melian and Zebib 2020) which was also observed in emotional tweets expressed on Twitter (Kohn 2020), a platform individuals use to convey their feelings (van Lent et al. 2017; Dubey and Tripathi 2020). Kohn (2020) shows that the average level of positivity expressed in tweets on remote work sank rapidly in the weeks before 11 March 2020, the day the WHO declared the COVID-19 a pandemic. However, from this day onwards, positivity toward remote work gradually rose again (see Fig. 1).

A possible explanation for this is that sociotechnical systems (STS) can be triggered to adapt to externally enforced changes and then realign people, technology, and processes (Carayon et al. 2015) to changed conditions until they reach a new stable state (Holling 1973; Vogus and Sutcliffe 2007). STS consist of two distinguishable but mutually interacting components: the technical and the social system (Bostrom and Heinen 1977; Bostrom et al. 2009). The pandemic induced a shift to remote work that represents an externally enforced change triggering the need of STS to realign both social and technical components and their interactions. Here, STS realignment refers to a major shift of communication and procedures to digital spheres to enable remote work (Kurland and Bailey 1999). Only if this realignment process leads to a state of high alignment to the changed conditions of remote work can we consider STS resilient. In other words, STS resilience is a state of high alignment to changed conditions following a realignment process. Since remote work is highly dependent on information and communication technologies (ICT) to interact with work peers and clients (Gajendran and Harrison 2007; Limburg and Jackson 2011) and requires a significant shift in communication and procedures to digital spaces (Kurland and Bailey 1999), it is likely that sentiments expressed in tweets, for example, mirror these kinds of ongoing realignments of STS.

So far, however, we lack evidence on the relationship between sociotechnical realignment and sentiments when describing experiences with remote work. Only if we understand why some organizations are more successful in realigning with a new situation will we foster resilience building as a resource for managing future disruptions (Fredrickson 2003; Boh et al. 2020). Accordingly, we explore how the realignment of STS affect subjects’ positive or negative sentiments when describing their experiences with an externally enforced shift to remote work. We address the following initial research question:

- How does the realignment of sociotechnical systems in times of an externally enforced shift to remote work affect positive or negative sentiments when describing working from home experiences?

It is also not yet clear how STS realignment relates to the resilience of humans embedded in STS. Personal digital resilience refers to how well an individual recovers from or adjusts to major disruptions such as the pandemic-induced transition to remote work (Boh et al. 2020; Kohn 2020). Our second research question is as follows:

- How does sociotechnical systems realignment in times of an externally enforced shift to remote work affect the personal digital resilience of employees embedded in STS?

We further want to investigate how an individual’s personal digital resilience relates to their feelings about an enforced shift to remote work. Hence, we address our third research question:

- Is personal digital resilience related to sentiments towards remote work?

**Fig. 2:**
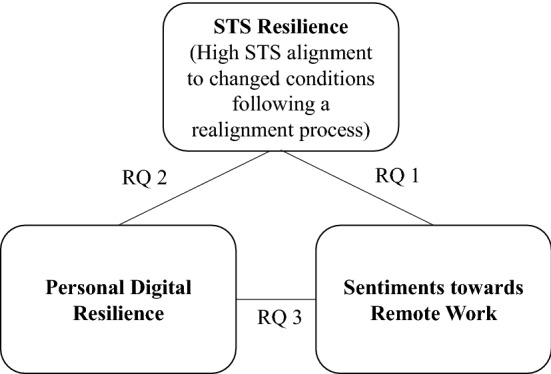
Research questions (RQ)

To answer these research questions illustrated in Fig. 2, we approached 40 test persons five months after the WHO declared COVID-19 a pandemic. We assume that five months is sufficient for STS to begin realigning to the new situation and to spawn a certain variety of alignment levels when comparing STS. Using the Q methodology (Stephenson 1968; Brown 1986; McKeown and Thomas 1988, 2013) allows us to split individuals into groups according to how well the STS they work and live with realigned to remote work. By deploying sentiment analysis (Hutto and Gilbert 2014), we hope to reveal differences in their sentiments about remote work, thereby augmenting our understanding of the relationship between STS alignment and sentiments (cf. RQ 1). Building on a resilience scale modified for the remote work context, we compare the personal digital resilience scores of individuals from both groups (cf. RQ 2) and the relationship to their sentiments about remote work (cf. RQ3).

The paper proceeds as follows: We first outline the STS framework as well as salient research on resilience in information systems and remote work to frame our study. We then describe our research design and, in particular, introduce Q methodology which we use to identify different levels of sociotechnical alignment with remote work, whereupon we present our results. Finally, we discuss our findings, limitations, and opportunities for future research.

## Related Research

### Sociotechnical Systems (STS) Framework

**Fig. 3:**
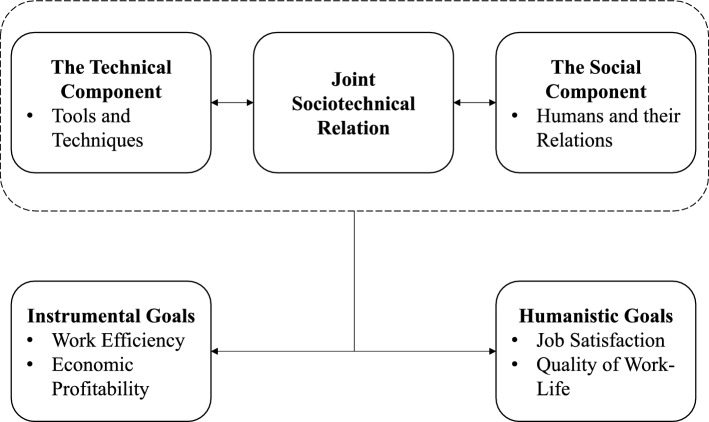
Sociotechnical perspective following Sarker et al. (2019)

As the theoretical foundation for our interpretation, we rely on the STS framework. For many academics, the sociotechnical perspective represents a key element of information systems (IS) research (Sarker et al. 2019), which has its origins in multiple post-World War Two studies that were designed to embrace improvements in working life (Trist and Bamforth 1951). In principle, STS consist of two distinguishable but mutually interacting components: the technical and the social system (see Fig. 3). The technical component comprises of tools and techniques needed to fulfill organizational tasks (Bostrom and Heinen 1977), while the social component is composed of employees and their attributes, such as skills, knowledge, or the social capital they bring to the work environment (Ryan et al. 2000; Bostrom et al. 2009). STS contends that it is the joint interaction of the two dimensions that is needed to achieve instrumental and humanistic outcomes, such as productivity or job satisfaction (Wallace et al. 2004; Bostrom et al. 2009). Sarker et al. (2019) argue the need to accept various socio-technical relationships within STS and point to a mutual, iterative and transformational nature of interactions, in which both the social and the technical play more than just an incidental or nominal role. It is through this interaction that humanistic and instrumental outcomes can be achieved in a synergistic manner. In other words, STS involve humans applying technologies to execute work tasks within an organizational environment to accomplish set goals (Bostrom and Heinen 1977; Carayon et al. 2015). Transferred to the pandemic-induced shift to remote work, employees apply technologies, such as online communication and collaboration tools, but in order to efficiently get their work done and improve their work life, a joint optimization of both components as well as their interaction are needed.

### Resilience in Information Systems (IS) Research

The concept of resilience first appeared in ecology (Holling 1973) and has gained considerable attention in natural and social sciences, operations management, psychology, and information systems (Allenby and Fink 2005; Annarelli and Nonino 2016). Resilience deals with accommodating external shocks and reaching a new stable system state (Holling 1973). Hence, it helps in coping with a broad array of disruptions and strains (Vogus and Sutcliffe 2007).

Research on resilience relevant to IS refers to adapting to changing constraints in the event of disturbances (Allenby and Fink 2005), thriving on changing circumstances (Fletcher and Sarkar 2013), and recovering from partial damage (Wang et al. 2010). Being resilient requires situational awareness related to IS, the management of IS vulnerabilities, adaptive capacity, risk intelligence, flexibility, and agility of IS in a dynamic environment (Sarkar et al. 2017). Resilience in IS literature is referred to as a trait, an outcome or a process capability (Sarkar 2017; Kohn 2020). We consider STS resilience to be a high degree of STS alignment as the outcome of a successful realignment process.

IS research with regard to resilience has three focal points: first, studies bringing humans and their capabilities to cope with disruptions into focus (Cho et al. 2007). Second, studies on resilient information systems (Smith et al. 2011). And third, studies on organizational resilience, marked by factors like the adaptability of information systems, agility, and flexibility (Erol et al. 2010). What is noteworthy is that the organization’s ability to build resilience is largely dependent on employee contributions, which in turn need to have recourse to organizational resources (Shin et al. 2012). This implies that both the technical system (tools, structures, and processes) as well as the social systems (employees including the capabilities and relationships with coworkers) need to be resilient to achieve STS resilience.

### Remote Work

Remote work (also referred to as telework, e-work, or virtual work) relates to a variety of flexible work arrangements where workers can operate from any location and beyond conventional office hours (Olson 1983; Stein et al. 2015; Wang et al. 2020). The success of distant working is highly dependent on information and communication technologies (ICT) to interact with work peers and clients (Gajendran and Harrison 2007; Limburg and Jackson 2011). It typically involves a significant shift of communication and procedures to digital spaces (Kurland and Bailey 1999).

Important research streams cover supervisory procedures (Olson 1983), communication difficulties (Kurland and Bailey 1999; Cramton 2001; Larson et al. 2020), social isolation (Clark et al. 2012; Wang et al. 2021), colliding work and non-work responsibilities (Golden et al. 2006; Wang et al. 2021), technostress (Ayyagari et al. 2011; Molino et al. 2020), and a lack of adequate IT infrastructure (Sarker and Sahay 2004).

While virtual working employees have full autonomy, managers lack all kinds of direct control (Olson 1983; Dimitrova 2003). Virtual workers generally communicate less frequently (Kraut et al. 2002), and it becomes more difficult for them to maintain deep and positive relationships with coworkers (Rice 1992; Gajendran and Harrison 2007). In the long run, social isolation is associated with less organizational identification, mainly because remote workers feel less respected (Bartel et al. 2012).

Remote work often reduces commute time and provides individuals with the opportunity to flexibly handle their work hours (Feldman and Gainey 1997). However, other studies show that the blurred boundaries between work and leisure time may create job stress and work-nonwork conflicts (Raghuram and Wiesenfeld 2004; Stein et al. 2015; Wang et al. 2021). This effect is greater for employees who regularly work at home (Raghuram and Wiesenfeld 2004). Another issue inherent to homeworking is an increase in family-related stress (Baruch and Nicholson 1997), technostress (Ayyagari et al. 2011), and work overload (Suh and Lee 2017).

Scholars have acknowledged that remote workers’ communication media use relates to experiencing stress, particularly email and face-to-face communication, videoconferencing, and instant messaging (Fonner and Roloff 2012). In addition, employees might feel uncomfortable using the video channel in online meetings (Stein et al. 2015). Besides, technical support has a positive impact on an employee’s satisfaction with flexible work arrangements (Haines III et al. 2002).

Over the years, researchers have reached a scientific consensus that remote work can only be successful if employees also have specific attributes, such as self-discipline (Olson 1983), mental readiness (Eckhardt et al. 2019), flexibility and self-management behavior (Haines III et al. 2002; Clark et al. 2012), self-efficacy (Raghuram and Wiesenfeld 2004), techno-affinity (Eckhardt et al. 2019) and emotional stability (Perry et al. 2018).

## Research Design and Methods

**Fig. 4:**
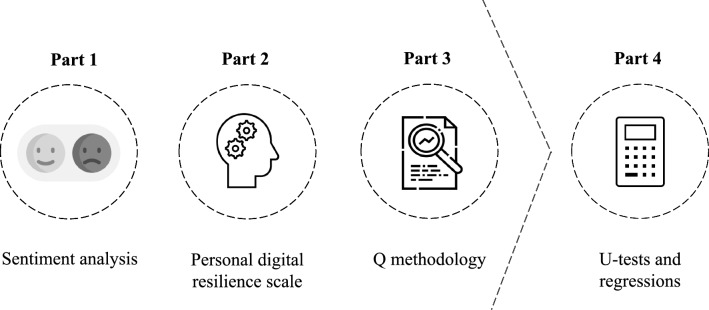
Research design

Our research design comprises four parts illustrated in Fig. 4: In the first part, we apply sentiment analysis to analyze employees’ positivity toward remote work. In the second part, we adjust a resilience scale to a remote work context to determine employees’ personal digital resilience scores. Third, we use Q methodology to identify groups of individuals whose STS are realigned significantly differently to remote work. This distinction into groups with different degrees of STS alignment serves as the foundation for subsequent analyses. In the fourth part we build on results of the previous steps and statistical tests to establish relations between STS alignment, sentiments and personal digital resilience in line with our research questions.

Next, sentiment analysis, the instrument to measure personal digital resilience, and Q methodology are outlined.

### Sentiment Analysis

To assess the participants’ attitudes toward remote work, we asked them to comment on how they felt during the shift to remote work at the beginning of the COVID-19 pandemic (11 March 2020), and how they feel about remote work currently (October 2020). We used free text entry masks to collect the respective statements. We received 80 statements overall, which means 100% of the participants delivered respective statements. The collected free texts allowed us to compute a sentiment score for each participant. We translated the German comments to English using DeepL,^1^ which draws on artificial intelligence to understand and translate texts. While the translation was mostly correct, we found minor grammatical errors. To minimize confounding the effects of incorrect translations,^2^ two German native speakers manually rated the DeepL translations of each statement as positive, neutral, or negative. Intercoder reliability using Cohen’s Kappa (Landis and Koch 1977) was 0.843.

We then performed sentiment analysis using the Python library VADER (Hutto and Gilbert 2014) and controlled the accuracy of the resulting compound score for each comment by comparing them to the manual ratings introduced above.^3^ We accepted all compound scores fitting the same category (positive ≥ 0.05, negative ≤ − 0.05, or neutral) as our manual rating. If the algorithm clearly misidentified a statement’s sentiment, we manually modified the respective score according to Domagalski (Domagalski 2020). Overall, we modified 19 out of 80 sentiment scores, which corresponds to an accuracy of 76.25% for the algorithm. Each modification is documented and justified in Online Appendix B.

### Personal Digital Resilience

We adapted the generic Employee Resilience Scale (Näswall et al. 2015) to the remote work context (see Table 1) to measure the participants’ personal digital resilience scores. The italic text passages indicate our modifications. Reliability and validity of the digital resilience scale are given: Cronbach’s alpha amounted to 0.771 after excluding item 9 for reliability purposes. Composite reliability exceeded the threshold of 0.7 (Hair et al. 2014) and supported the construct's internal consistency.

**Table 1:** Personal digital resilience scale

Seven-point Likert-type scale, ranging from 1 (Never) to 7 (Almost always)	
Context: Transition into remote work during the COVID-19 pandemic	
1	I effectively collaborate with others to handle unexpected challenges *while working remotely*
2	*While working from home*, I successfully manage a high workload for long periods of time
3	I resolve crises competently *when working from home*
4	I learn from mistakes *when working from home* and improve the way I do my job
5	I re-evaluate my performance and continually improve the way I do my work *from home*
6	I effectively respond to feedback *about my remote work*, even criticism
7	I seek assistance for *remote* work when I need specific resources
8	I approach managers *through digital channels* when I need their support
9	I use the change *toward working from home* as an opportunity for growth

### Q Methodology

Q methodology allows for the identification of individuals who share the same notions, opinions, or beliefs (Stephenson 1968; Brown 1986; McKeown and Thomas 1988, 2013). It offers a systematic vehicle for studying human subjectivity. Researchers select representative statements from the concourse—also referred to as the Q sample—and then ask participants to express their agreement or disagreement with the items in the sample, thereby producing a Q sort. A Q sort represents an individual’s personal viewpoint on the given matter. Since all Q sorts are correlated, they produce an N x N correlation matrix, which can be used for factor analysis to condense the statements into a few meaningful factors. The resulting factors represent individuals who share similar viewpoints.

Overall, Q methodology involves the subjective communicability of individuals’ points of view that are nonsubstantive (Stephenson 1968) and thus self-referential (Brown 1986). Individuals’ subjective responses all taken together establish a concourse of communication, which constitutes the input for Q studies. Commonly, Q methodology encompasses five steps: first, representative statements reflecting the concourse on the matter at hand are collected (Q sample). This is followed by the selection of participants, which then, in step 3, model their individual viewpoints by sorting items according to a given pattern (Q sorts). Step 4 includes analyzing the correlation matrix and calculating factor loadings and factor scores. It is important to note that resulting factors represent groups of participants that share similar viewpoints. A factor loading is, thus, the degree to which a participant belongs to a group and the factor scores show how much a group concurs with each statement (Zabala 2014). Their interpretation takes place in the last step (McKeown and Thomas 2013). In the following, we will elaborate further on the essential steps.

## Analyzing STS Alignment to Remote Work

We apply Q methodology to identify groups of individuals whose STS realigned to different degrees to remote work. This distinction serves as the basis from which we subsequently establish the interplay of STS resilience (i.e., a high STS alignment to the changed conditions of remote work), sentiments and personal digital resilience.

### Step 1: Composing the Q Sample

The composition of Q samples commences with collecting a wide range of views on the subject matter; here, the shift to remote work during the COVID-19 pandemic. The most preferred way to compose Q samples is through interviews because they allow self-referential and subjective viewpoints to be revealed (McKeown and Thomas 2013). We conducted 12 in-person interviews to capture various viewpoints on the enforced transition to remote work that comprehensively represent the concourse. Participants were randomly selected following a call for people who shifted to remote work at the beginning of the COVID-19 pandemic. To minimize response bias, we emphasized that participation was anonymous, confidential, and voluntary (Podsakoff et al. 2003). All interviews were conducted by the same researcher (Brod et al. 2009) to maintain consistency. Two-thirds of the respondents were female (Table 2). The average interviewee was 40.42 years old and had spent 6.88 years in their current job. All participants graduated at least from high school and most reported having a university degree. Five interviewees stated they lived alone, and seven shared their home with a respective partner, family, or flatmates.

**Table 2:** Demographics of the interviewees

#	Gender	Age	Time in current job (in years)	Job title	Living situation
1	f	31	3	Social media analyst	With parents, two siblings
2	f	28	1	Business controller	With one sibling
3	f	29	1	Senior controller	With parents
4	f	26	0.5	Junior creative solutions manager	Flat sharing with seven other occupants
5	f	26	2	Business development advisor	With partner
6	f	50	5	Senior manager	Alone
7	m	58	20	Software engineer	Alone
8	m	25	1	Software engineer	Alone
9	m	55	23	Development engineer	With wife and child
10	m	36	1	Payroll coordinator	With wife and child
11	f	59	10	Service expert billing	Alone
12	f	62	15	Human resources administrator	Alone

The interviews lasted between 12 and 45 min, were recorded and subsequently transcribed. A semi-structured format was used (Myers and Newman 2007). We followed a prepared guideline, which consisted of three parts: the first part addressed the demographic characteristics of the interviewees. Second, we collected insights concerning the interviewees’ experience with the suddenly induced shift to remote work. We explicitly asked the participants to report their experiences with remote work during the pandemic, in particular, difficulties arising from the transition to remote work, perceived advantages and disadvantages of working from home, perceived changes in productivity and communication, perceived organizational support, and the specifics of their remote work arrangements. Third, we investigated the interviewees’ personal digital resilience (Sarkar 2017) and asked about their situation awareness, flexibility, agility as well as their ability to deal with vulnerabilities, how they anticipate information security risks, and how they adapt to changes in the work environment.

An in-depth analysis of the interviews following an inductive coding procedure (Mayring 2010) unveils various aspects that might drive or delay STS realignment to remote work conditions, which we matched to related literature. We compiled the Q sample as follows (Barchak 1979): We screened the relevant articles identified in our literature review and our interview transcripts for adequate items. We identified the following items as relevant for the Q sample: (1) team support in terms of frequency and intensity of formal and informal contact to colleagues (Waizenegger et al. 2020), (2) management support (Sakurai and Chughtai 2020; Wade and Shan 2020), (3) technical support and training (Asatiani et al. 2020), (4) security concerns (Naidoo 2020), (5) performance (Waizenegger et al. 2020), (6) technostress (Ayyagari et al. 2011), (7) work-home conflict (Benlian 2020), (8) self-efficacy (Wang and Haggerty 2011), (9) digital well-being (Eckhardt et al. 2019), and (10) personal beliefs, like opinions on video conferencing or the future of remote work. Next, the feedback of a focus group (three participants) evaluating an initial set of statements was incorporated in the specification of the final Q sample.

The final Q sample comprises 40 statements, which is well in the middle of the range of 20 to 60 statements assessed as meaningful for Q samples (Donner 2001).^4^ To operationalize the STS framework, we matched each Q sort statement to its most fitting category of the STS framework (online Appendix A). For instance, statements matched to the social component address team and management support, while those in the technical component refer to equipment, software and tools. The joint component encompasses statements referring to the interplay of social aspects and technologies, such as information security behavior or training and workshops on tools and techniques. All statements dealing with personal beliefs, technostress, work-home conflict and digital well-being fit with the humanistic component of the STS framework. Instrumental goals are reflected, for instance, in statements on perceived performance. The matching was carried out independently by two researchers with a high intercoder reliability of 0.897 using Cohen’s Kappa (Landis and Koch 1977). Following the revelation of which of the potential aspects contained in the statements actually distinguish different degrees of STS alignment through data analysis in step 4, this classification will assist factor interpretation in step 5.

### Step 2: Selection of Participants

The Q methodology aims to investigate key opinions of selected participants, instead of studying large nonrandomized participant samples as done in conventional studies in R methodology^5^ (Dziopa and Ahern 2011). It is, therefore, neither necessary nor recommended to engage with a large group of participants but rather to seek a 1:1 ratio of statements to participants (Watts and Stenner 2005). Since our Q sample consists of 40 statements, we approached a total of 43 participants, around half of them via the German-based premium panel agency ClickWorker^6^ and half of them via the researchers’ personal networks. In doing so, we tried to avoid sampling bias and guaranteed extensive variability in participants’ attitudes to remote work (Brown 1980). The demographics of participants are summarized in Table 3. While we made an enforced shift to remote work due to the pandemic a prerequisite for participation, we do not know how well each participant’s STS realigned to the new remote work conditions at the time of the respective participant’s recruitment. We will only identify this in step 4.

**Table 3:** Demographics of participants

	Total
Gender	
Male	20
Female	20
Age	
20 or younger	1
21–29	10
30–39	14
40–49	6
50–59	4
60–69	4
70 or older	1
Highest education	
High school diploma	14
Bachelor’s degree	7
Master’s degree	19
Living situation	
Alone	15
With one other person	13
With multiple others	12
Working situation	
Employed for wages (part-time)	10
Employed for wages (full-time)	25
Self-employed	5
Transition to remote work due to COVID-19	
Partly	17
Fully	23

### Step 3: Q Sort

**Fig. 5:**
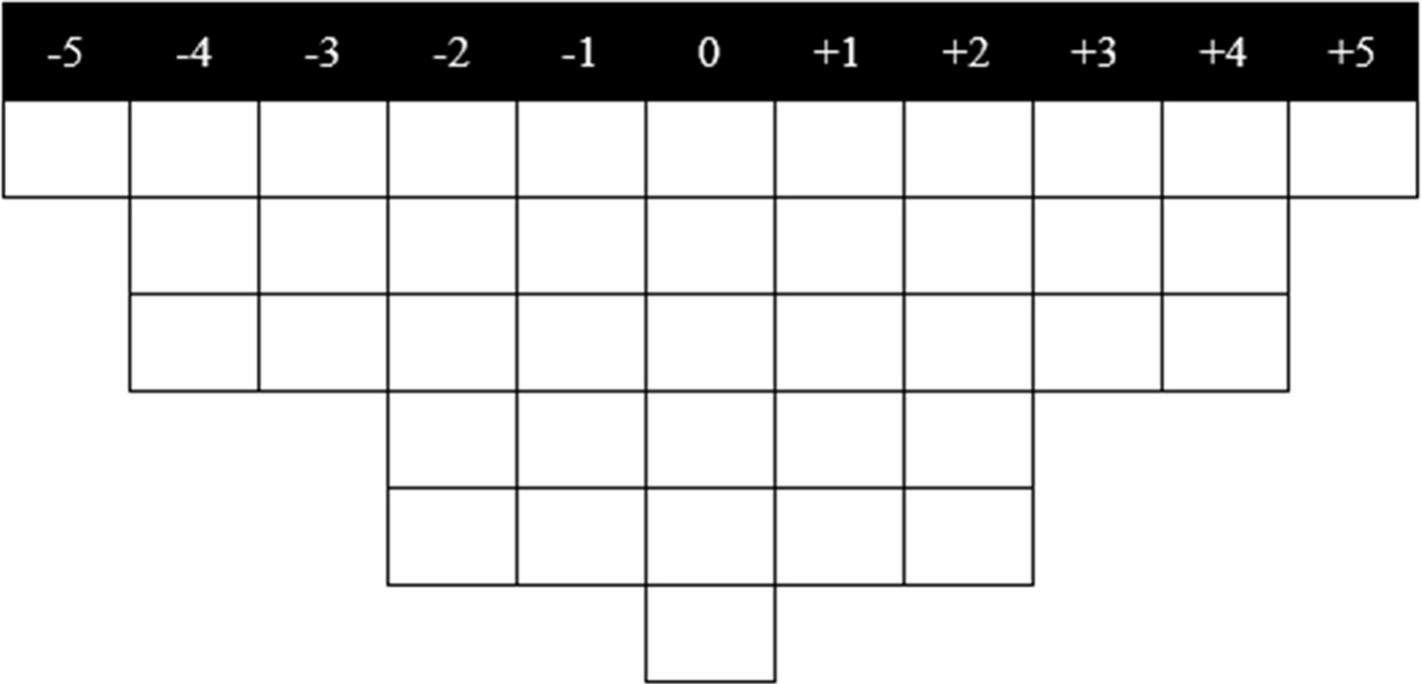
Response grid for Q sorting

Q sorting involves modeling Q sample items along a given continuum. Participants serve as sorters and evaluate all items by comparing them to all other items. They then arrange the items according to a compulsory distribution, like the one shown in Fig. 5 (McKeown and Thomas 2013).

To foster familiarity with all 40 statements, we first asked participants to place each statement in one of three piles: one for statements they agree with, one for statements they disagree with, and one for the remaining statements they feel neutral or uncertain about (McKeown and Thomas 2013). All items were displayed in random order. Next, participants were asked to sort all 40 statements according to their degree of agreement from − 5 (no agreement at all) to + 5 (full agreement). In Q methodology, participants are typically asked to place the statements in a quasi-normal distribution shape consisting of 11 to 13 rating categories depending on the size of the Q sample (Brown 1980; Watts and Stenner 2005). While the distribution shape does not substantially affect statistical analysis, it encourages participants to systematically sort the statements (McKeown and Thomas 1988). Figure 5 illustrates the response grid we applied and implemented in a web-based interface. It fits 40 ratings to a continuum of 11 rating categories from − 5 to + 5, including 0. Only a few ratings are placed at the extreme ends (− 5 to − 3 respectively + 3 to + 5).

We used a web-based interface (Aproxima 2015) and a PHP-backend (Hackert and Braehler 2007) to collect the Q sorts online, an easy and user-friendly approach. The data collection took place from 23 October 2020 to 29 October 2020. Beforehand, the respondents were informed about the study’s objective to analyze employees' experiences with remote work during the pandemic. We eliminated participants who did not finish the study or showed unduly quick response times. A total of 40 out of the 43 collected data sets were kept for further analysis.

### Step 4: Data Analysis (Q Methodology Results)

Data analysis encompasses the consecutive application of three statistical procedures: first the correlation followed by factor analysis and eventually computing factor scores which are needed for factor interpretation (McKeown and Thomas 2013). To analyze the Q sorts, calculate factor loadings and factor scores, we used the R package “qmethod” (Zabala 2014). We revealed two factors and considered factor loadings above a critical value of ± 0.31 as significant (Brown 1980). This value is at the lower limit of the range of ± 0.31 (*p* < 0.05) to ± 0.41 (*p* < 0.01) when analyzing 40 statements in the Q sample (Watts and Stenner 2005). Significant loadings on factors 1 and 2 are flagged in Table 4.

**Table 4:** Overview of the significant Q sort loadings

Parti-cipant	Q-sort loadings on Factor 1	flag	Q-sort loadings on Factor 2	flag	Parti-cipant	Q-sort loadings on Factor 1	flag	Q-sort loadings on Factor 2	flag
1	0.62	*	− 0.35		21	0.38	*	− 0.23	
2	0.64	*	0.28		22	0.18		0.60	*
3	0.49	*	0.10		23	0.40	*	0.24	
4	− 0.02		0.67	*	24	0.73	*	0.06	
5	0.17		0.65	*	25	− 0.09		0.69	*
6	0.79	*	0.06		26	0.61	*	0.24	
7	0.05		0.65	*	27	0.03		0.39	*
8	0.34		0.36	*	28	0.26		0.72	*
9	0.48	*	0.20		29	0.73	*	− 0.39	
10	0.51	*	0.05		30	0.71	*	0.15	
11	0.02		0.40	*	31	0.80	*	0.09	
12	0.16		− 0.15		32	0.49	*	0.31	
13	0.64	*	− 0.11		33	0.41	*	0.00	
14	0.43	*	0.42		34	0.74	*	0.15	
15	0.76	*	0.11		35	0.60	*	0.27	
16	0.31		0.39	*	36	0.64	*	0.12	
17	0.62	*	0.08		37	0.74	*	− 0.23	
18	0.11		0.62	*	38	− 0.10		0.21	
19	0.30		0.64	*	39	0.71	*	0.20	
20	0.37	*	0.17		40	0.41	*	0.35	

*Q sort loading with critical value ± 0.31 and no confounding loading on the other factor. The calculations are based on Brown (Brown 1980)

The two factors yielded by Q methodology represent two groups of people: 26 Q sorts load significantly on factor 1, meaning that 26 participants belong to the first group, while 12 Q sorts load significantly on factor 2. We excluded two participants due to a lack of significant loadings to any factor (Watts and Stenner 2005; Dziopa and Ahern 2011). For interpretation purposes, we aim to concentrate on (1) the high and low statement rankings focusing on the *distinguishing statements for both factors*, and on (2) the statements’ high absolute z-scores since they mirror the *relationship between statements and factors*. Z-scores specify how much each factor concurs with a statement (Zabala 2014). They are the weighted averages of the scores given by the Q sorts that were flagged to a statement (Zabala 2014). Table 5 displays the Z-scores as well as the distinguishing and consensus statements. In step 5, we will elaborate on how these core statements separate the two identified groups.

**Table 5:** Factor Z-score ranks and absolute differences in Z-scores between factors

State-ment	Factor 1		Factor 2		Abs. diff	State-ment	Factor 1		Factor 2		Abs.diff
	z-score	Rank	z-score	Rank			z-score	Rank	z-score	Rank	
1^d^***	1.06	7	− 0.4	27	1.47	21^c^	1.24	6	1.55	3	− 0.31
2^d^***	0.47	12	− 1.0	30	1.50	22^d^***	− 0.9	34	0.64	12	− 1.51
3^c^	− 0.6	32	− 0.6	28	− 0.03	23^d^***	1.57	4	0.02	21	1.55
4^c^	0.1	18	0.32	16	− 0.22	24^d^***	− 1.0	36	0.82	9	− 1.79
5^c^	− 0.2	21	− 0.3	24	0.09	25^d^***	− 0.4	26	1.79	2	− 2.21
6^d^***	− 0.3	22	− 1.4	36	1.04	26^d^**	− 0.4	24	0.24	19	− 0.62
7^d^**	− 0.6	31	− 1.4	38	0.76	27^d^***	− 2.2	40	− 1.1	32	− 1.10
8^c^	− 1.4	37	− 1.1	33	− 0.32	28^c^	0.44	13	0.31	17	0.12
9^d^***	− 0.6	29	0.57	14	− 1.13	29^d^***	− 0.4	27	1.03	7	− 1.45
10^d^**	− 0.7	33	− 1.5	39	0.79	30^c^	− 0.5	28	− 0.3	23	− 0.25
11^c^	1.49	5	1.24	5	0.25	31^d^*	0.5	11	0.92	8	− 0.42
12^d^**	0.53	10	− 0.3	25	0.84	32^c^	0.56	9	0.62	13	− 0.06
13^d^*	0.37	14	− 0.0	22	0.38	33^d^*	0.13	17	0.55	15	− 0.42
14^d^***	0.29	15	− 0.7	29	1.03	34^d^***	1.01	8	0.13	20	0.88
15^d^***	− 0.00	19	− 1.6	40	1.57	35^d^**	− 0.6	30	0.25	18	− 0.81
16^d^***	− 0.4	23	− 1.4	37	1.02	36^d^***	− 0.4	25	1.18	6	− 1.59
17^d^***	0.28	16	− 1.2	35	1.51	37^d^***	− 1.6	38	0.7	11	− 2.32
18^d^***	1.83	2	− 1.2	34	2.99	38^c^	1.78	3	1.97	1	− 0.19
19^d^***	− 0.9	35	0.7	10	− 1.60	39^c^	− 0.1	20	− 0.4	26	0.27
20^d^**	− 1.6	39	− 1	31	− 0.58	40^d^**	2.19	1	1.36	4	0.83

^c,^^d^Marks the distinguishing (with significance thresholds for *p < 0.05, **p < 0.001, and ***p < 0.000001) and consensus statements, based on the absolute differences between factor Z-scores being larger than the standard error of differences (Brown 1980; Zabala 2014)

### Step 5: Factor Interpretation

We use the five core components of the sociotechnical framework to assess the most important and distinguishing statements based on the factor Z-scores and the ranks in Table 5. Table 6 summarizes our interpretation of the Q methodology results leading to two different groups of people: employees working remotely in highly aligned STS (“high STS alignment group”) and employees working remotely in STS with low degrees of alignment (“low STS alignment group”). Below, we summarize our characterization for each group and highlight important interpretative steps justifying our interpretation.

**Table 6:** Outline of the results

STS framework component	Factor 1: High STS alignment group	Interpretation	Factor 2: Low STS alignment group	Interpretation
Social component	Statement 1 (rank 7, z: 1.06)	Positive → High degree of social support		
Technical component	Statement 18 (rank 2, z: 1.83)	Positive → High degree of organizational tech support		
Joint sociotechnical relation	Statement 22 (rank 34, z: − 0.9)	Positive → Self-confidence with respect to security	Statement 15 (rank 40, z: − 1.6)	Negative → Low degree of organizational support
	Statement 19 (rank 35, z: − 0.9)	Positive → Self-confidence with respect to security	Statement 17 (rank 35, z: − 1.2)	Negative → Low degree of organizational support
Humanistic goals	Statement 37 (rank 38, z: − 1.6)	Positive → Positivity toward technological change	Statement 25 (rank 4, z: 1.79)	Negative → High work-home conflict
			Statement 29 (rank 7, z: 1.03)	Negative → Perceived privacy invasion
			Statement 36 (rank 6, z: 1.18)	Negative → Uneasiness when using technology
Instrumental goals	Statement 23 (rank 4, z: 1.57)	Positive → High perceived performance		
	Statement 24 (rank 36, z: − 1)	Positive → High degree of self-efficacy		

The high STS alignment group exhibits cohesion across all sociotechnical dimensions. We observe an even emphasis on the social and the technical ends, especially on the interplay of these two components. The social and technical conditions of work interact in a way that humanistic and instrumental outcomes are balanced. For instance, employees in the high STS alignment group receive social and technical support, which together build confidence in their ability to interact with technologies and adhere to security guidelines in a virtual environment. The positive outcomes emerging from this interaction are both instrumental (e.g., a higher performance) and humanistic (e.g., a positive attitude towards technological change). Hence, communication and procedures were successfully shifted to digital spheres to enable remote work in line with our definition of STS realignment. We conclude that the transformations of the STS towards the enforced integration of remote work for the high STS alignment group are advanced. This state of high alignment to changed conditions following a successful realignment process reflects a high STS resilience.

Members of the high STS alignment group understand the need to switch to remote work and agree that such work arrangements will play an essential role in an increasingly digitalized world (statement 40, rank 1, z = 2.19). Their sociotechnical characteristics are as follows:

- Social component: To compensate for not meeting their coworkers face-to-face and not having the opportunity to engage in watercooler chats, these employees intensify virtual contacts with coworkers (statement 1, rank 7, z = 1.06).
- Technical component: These employees receive a high level of organizational support. For instance, they have access to technical equipment necessary to successfully transition from working at an office to working at home (statement 18, rank 2, z = 1.83).
- Joint sociotechnical relation: They are eager to keep the company safe and adhere to security guidelines. For instance, they do not use the pandemic as a justification to defy security rules (statement 19, rank 35, z-score: − 0.9). As a result, they do not worry much about security (statements 22, rank 34, z = − 0.9).
- Humanistic objectives: They understand the need to switch to remote work; they even feel comfortable with the idea of living in a digitalized world (statement 37, Rank 28, z = − 1.6).
- Instrumental objectives: These employees efficiently manage the change to digital communication, easily handle disruptions of work routines (statement 23, rank 4, z = 1.57), and perceive remote work as a means of learning new things at work (statement 24, Rank 36, z = − 1.0).

The low STS alignment group lacks social and technical characteristics. In contrast to the high STS alignment group, they do not receive the technical equipment and tools needed to execute their work tasks from home (statement 18, rank 34, z = − 1.2). Moreover, their needs for social interaction with coworkers remain unfulfilled (statement 1, rank 27, z = − 0.4). Neither procedures nor communication were optimized for digital spheres, which is in contrast to our definition of a successful STS realignment. When the poorly aligned social and technical components interact, it is hard to achieve the desired humanistic or instrumental outcomes under remote work conditions. For instance, productivity decreases since employees embedded in these STS feel remote work to be a barrier to learning new things (statement 24, rank 9, z = 0.82). It is hardly surprising that accomplishing a stable state after the externally induced realignment of the STS is a distant prospect. Hence, these STS cannot be considered resilient.

The low STS alignment group has trouble adapting to the digitalized work conditions when transitioning to remote work during the COVID-19 pandemic. Their characteristics are as follows:

- Humanistic objectives: To start with, these employees tend to feel their privacy privacy by unscheduled work calls after working hours (statement 29, Rank 7, z = 1.03). Turning on their cameras in large online meetings makes them feel uncomfortable (statement 36, Rank 6, z = 1.18). Furthermore, they report high levels of technostress and find it challenging to manage nonwork-related sources of stress while working from home (statement 25, Rank 4, z = 1.79).
- Joint sociotechnical relation: They receive significantly less organizational support. Most importantly, their companies do not provide them with the necessary workshops on data protection or IT security (statement 15, Rank 40, z = − 1.6), and their organizations do not offer tool training (statement 17, Rank 35, z = − 1.2).

For the sake of completeness, we controlled for gender, age, education, living situation, type of employment, as well as the exact month of transitioning to remote work during the pandemic. We find a significant difference in the marital status between the groups *(Fisher’s Exact Test, p* = *0.044)* (Fisher 1939; Sauro and Lewis 2012) with the majority of individuals in the high STS alignment group being in a relationship or married and the majority of those in the low STS alignment group being single. The other control variables show no significant impact (see online Appendix C).

## Results

As shown above, the levels of realigning sociotechnical systems (STS) in times of an externally induced shift to remote work differ significantly: while the high STS alignment group experience a harmonized interplay of social and technical components, the low STS alignment group lack not only technical and social support but also a harmonized interaction between social and technical components. We build on the results of our sentiment analysis, the personal digital resilience scores and the differentiation between low and high STS alignment to apply statistical analyses aimed at answering our research questions. Specifically, we apply U-tests and regression analyses to test if the dependent variables (DV) sentiments towards remote work and personal digital resilience are correlated with the independent variables (IV) of loadings on factor 1 and 2 (i.e., how much each participant loads on the high vs. low STS alignment groups).

**Fig. 6:**
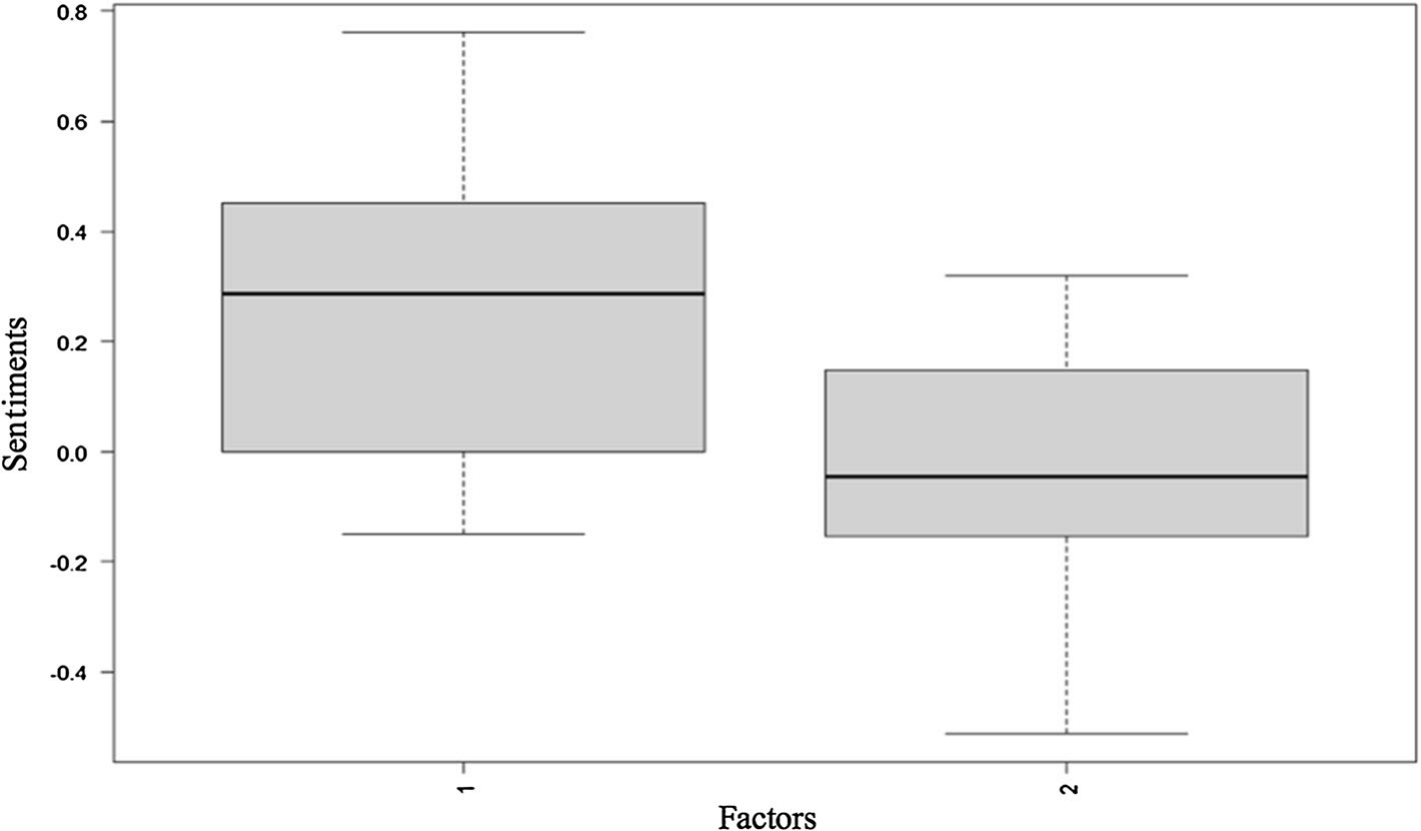
Box plot median sentiments ~ factors

To answer our first research question (How does sociotechnical systems realignment in times of an externally enforced shift to remote work affect positive or negative sentiments when describing working from home experiences?), we compare sentiments of participants who loaded more significantly on factor 1 (i.e., the high STS alignment group) with sentiments of those who loaded stronger on the second factor (i.e., the low STS alignment group), which reveals the following: the median compound sentiment scores of the high STS alignment group *(0.286)* is significantly higher than the median compound sentiment scores of the low STS alignment group *(*− *0.044) (Mann–Whitney U-Test, p* < *0.004, medium effect size r* = *0.427*) (see Fig. 6). This indicates a significant difference in how people feel about remote work depending on how successfully their STS realigned to the new remote work conditions. Employees working in highly aligned, thus resilient, STS feel and communicate significantly more positively about their remote work experiences.

**Fig. 7:**
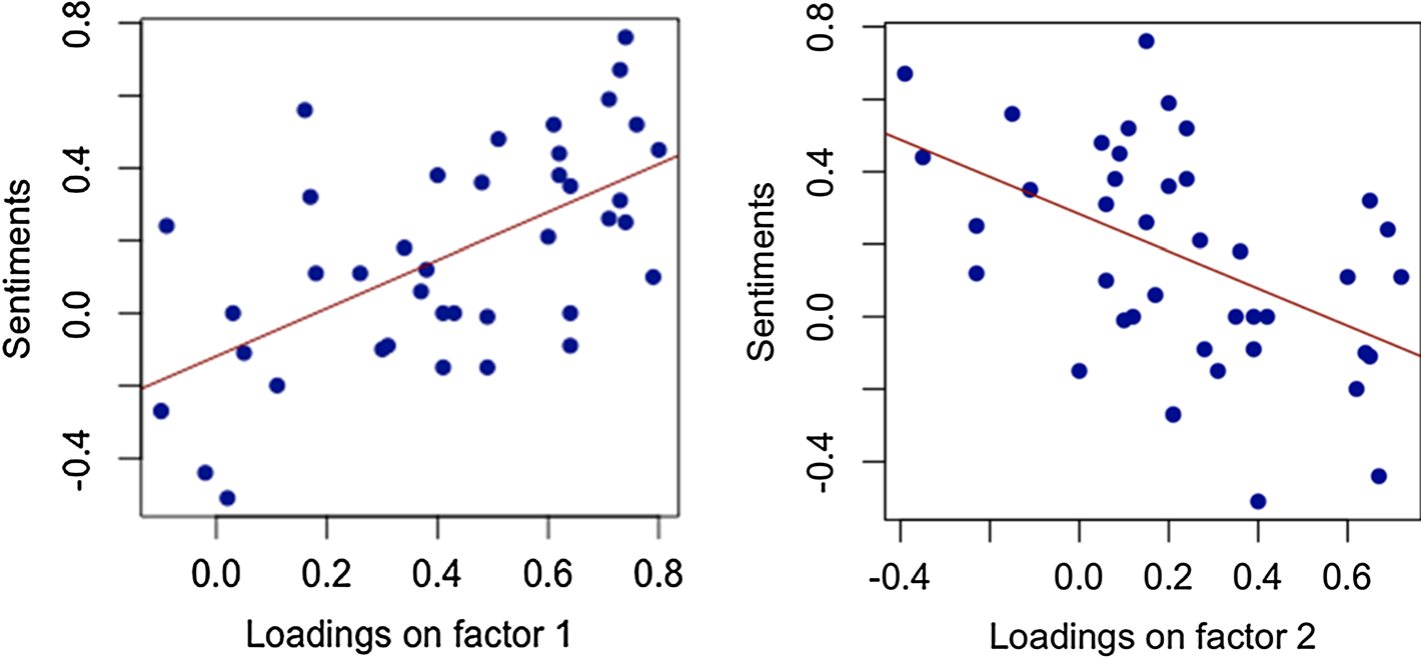
Regression sentiments ~ factors

Linear regression analysis confirms a statistically significant relation of sentiments toward remote work (DV) and loadings on factor 1 (IV) (*ß* = *0.66, p* < *0.001, R*^*2*^ = *0.353*). The higher a participant loads on factor 1, the more positive their sentiments towards remote work are. Similarly, linear regression analysis confirms a statistically significant relation of sentiments toward remote work (DV) and loadings on factor 2 (IV) (*ß* = − *0.51, p* = *0.001, R*^*2*^ = *0.248*). The higher a participant loads on factor 2, the less positive their sentiments towards remote work are. Interestingly, the correlations of sentiments with the high STS alignment group (factor 1) and the low STS alignment group (factor 2) are opposite in sign as illustrated in Fig. 7. Analysis of the assumptions of linear regression showed no issues (see online Appendix D).

**Fig. 8:**
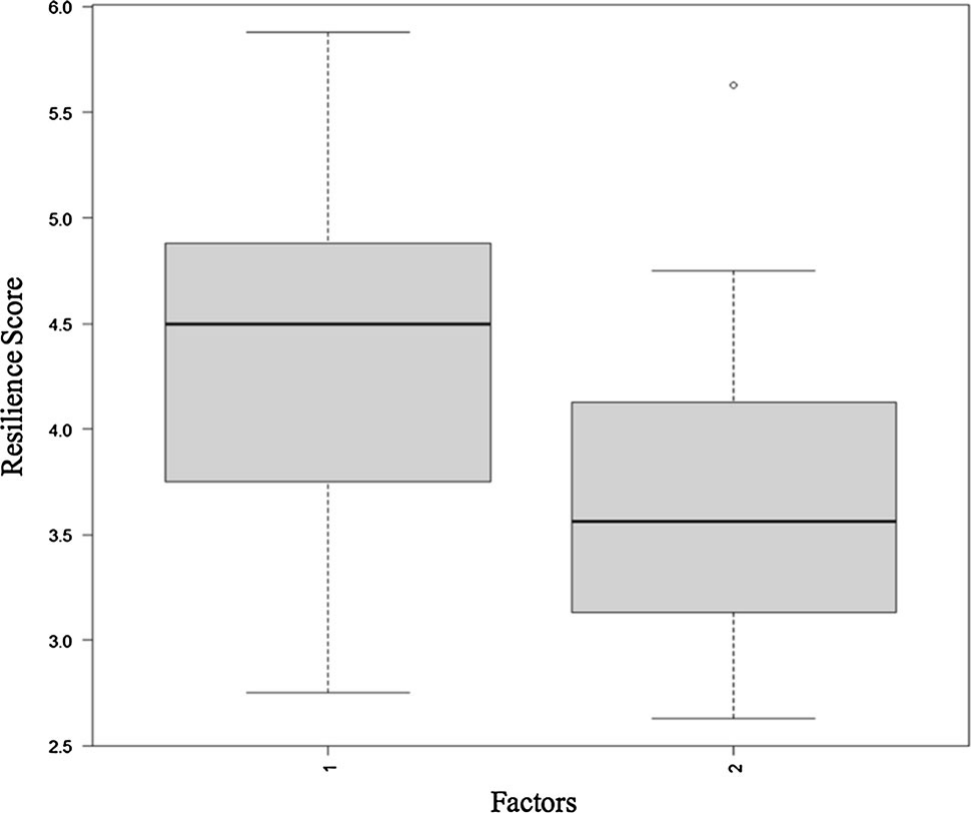
Box plot median resilience score ~ factors

In line with our second research question (How does sociotechnical systems realignment in times of an externally enforced shift to remote work affect the personal digital resilience of employees embedded in STS?), we compared the median personal digital resilience scores of the high STS alignment group (4.500) with the median personal digital resilience scores of the low STS alignment group (3.565) and found a significant difference (*Mann–Whitney U-Test, p* < *0.022, medium effect size r* = *0.325*) (see Fig. 8). We thereby show a significant positive relationship between STS resilience in the form of a high alignment to changed conditions and personal digital resilience.

**Fig. 9:**
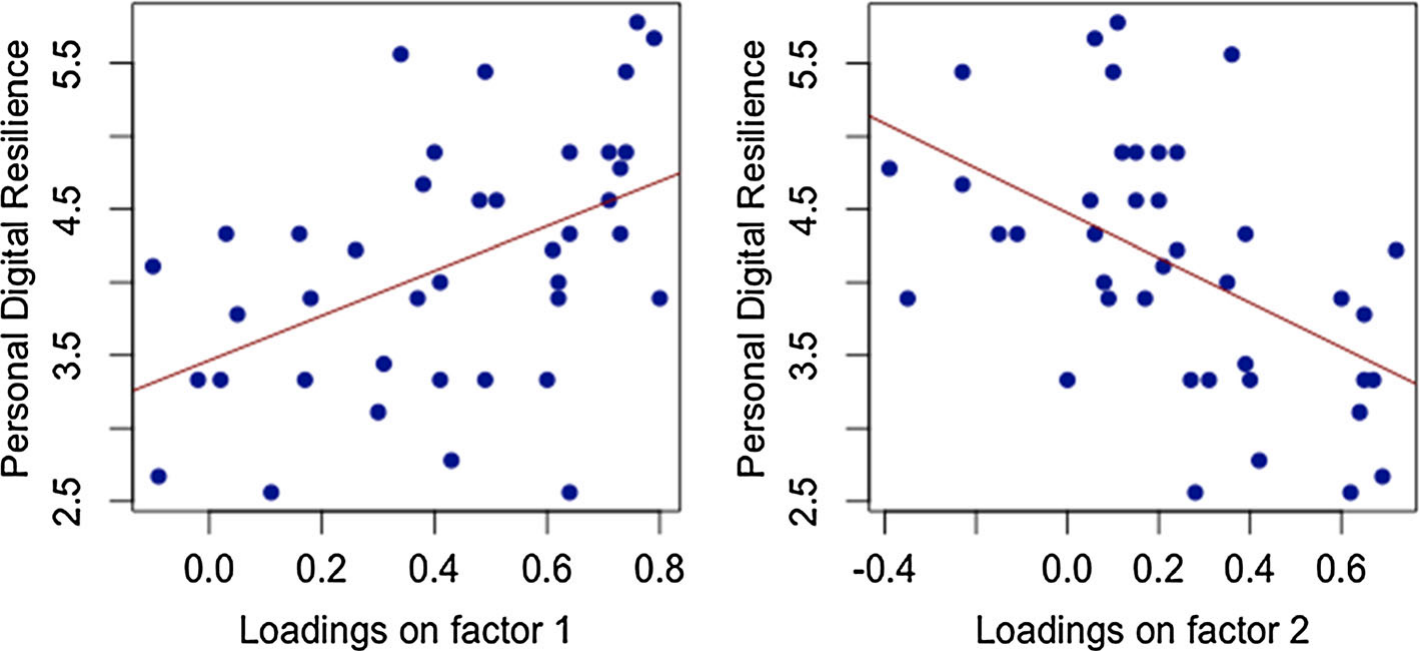
Regression personal digital resilience ~ factors

Linear regression analyses confirm a statistically significant relation of employees’ personal digital resilience scores (DV) and loadings on factor 1 (IV) (*ß* = *1.54, p* = *0.002, R*^*2*^ = *0.227*) as well as on factor 2 (IV) (*ß* = − *1.54, p* = *0.001, R2* = *0.266*). The higher a participant loads on factor 1, the higher their personal digital resilience scores. This means the stronger a participant belongs to the high STS alignment group, the more personal digital resilience they have. Similarly, the higher a participant loads on factor 2, the lower their personal digital resilience scores (see Fig. 9). Analysis of the assumptions of linear regression showed no issues (see online Appendix E).

**Fig. 10:**
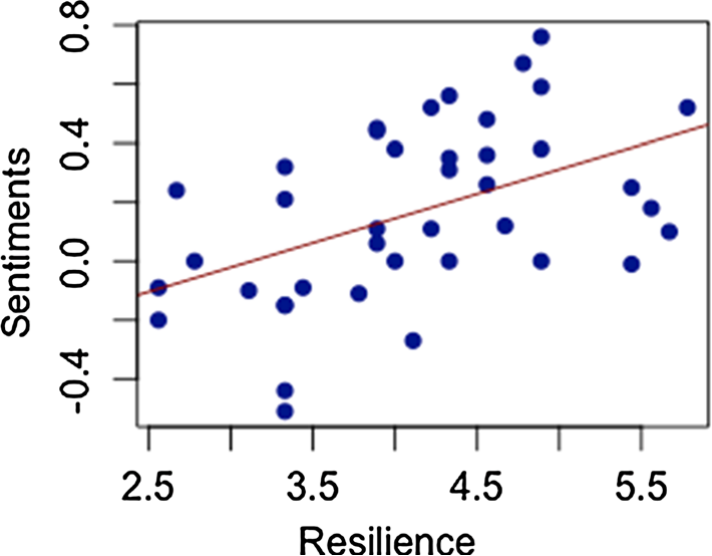
Regression sentiments ~ personal digital resilience

For our third research question (Is personal digital resilience related to sentiments towards remote work?), we compared participants’ personal digital resilience scores to their sentiments expressed toward remote work. Specifically, we aimed at validating the relationship between participants’ sentiments toward remote work and their ability to handle unexpected digital challenges and thrive in times of crisis. Linear regression analysis confirmed a statistically significant relation of positivity toward remote work (DV) and personal digital resilience scores (IV) (*ß* = *0.166, p* = *0.002, R*^*2*^ = *0.230*), see Fig. 10. Analysis of the assumptions of linear regression showed no issues (see online Appendix F).

To summarize, we found: (1) Positivity toward remote work is significantly higher for the high STS alignment group when compared to the low STS alignment group. (2) Personal digital resilience is significantly higher for the high STS alignment group when compared to the low STS alignment group. (3) Positive sentiments toward remote work are positively related to personal digital resilience.

## Discussion

The Covid-19 pandemic has raised global awareness of the importance of building digital resilience so as to better adjust to pandemic-related disruptions and be prepared for future exogenous shocks, which are expected to increase in frequency and severity as the twenty-first century progresses (Heeks and Ospina 2018; Boh et al. 2020). One example of how information technology can be leveraged to battle the negative consequences of the pandemic and achieve resiliency is remote work. Our study contributes to the understanding of when an externally triggered transition to remote work leads to a resilient STS and when it does not, specifically which aspects determine whether or not a realignment process of moving communication and procedures to digital spheres is successful. Our study’s objective was to further explore how the successful or unsuccessful STS realignment manifests itself on an individual level in terms of personal digital resilience and positivity or negativity in sentiments when describing experiences with the externally enforced shift towards remote work as well as their personal digital resilience. This investigation into resilience on both the STS level and the individual level is an important endeavor towards a gaining deeper understanding of the complex yet understudied phenomenon of digital resilience. Understanding how these mechanisms work with regard to remote work in the current pandemic will allow STS and individuals to strengthen their resilience against future shocks. To this end, we approached 40 test persons five months after the WHO declared COVID-19 a pandemic. By first deploying Q methodology (Stephenson 1968; Brown 1986; McKeown and Thomas 1988, 2013), we identified two groups of people who are significantly different with respect to their subjective views on remote work and the sociotechnical systems they work and live with; the first group exhibits a high degree of sociotechnical systems alignment to the externally enforced remote work situations, while the second group lives and works in a low STS alignment to the externally enforced remote work situations. Q methodology ensures the significant difference of both alignment levels.

Based on this distinction—which represents our first contribution—we are able to derive recommendations for organizations and employers on which aspects drive STS realignment to be successful, leading to high STS resilience. For instance, the degree of STS resilience can be improved by fostering social support amongst colleagues, for instance by encouraging daily video or voice calls and by strengthening employees’ self-confidence with respect to security through tailored information security policies for the work from home context. Other strategies to improve STS resilience during turbulent times such as the Covid-19 pandemic is to implement interventions targeting the self-efficacy of employees and the building of a positive attitude toward technological change. STS resilience is also higher when employees are reminded of the silver linings that come along with having to deal with a difficult disruption, in our case the increased productivity and reduced commute time when working from home. On the other hand, we identified aspects delaying the STS alignment that should be avoided. These include providing little organizational support such as tool or data protection trainings, allowing a high work-home conflict and privacy invasions, as well as not addressing employee at unease when using technologies.

Next, we explored if a higher level of STS alignment is related to a higher sentiment score of humans embedded in this STS. Using sentiment analysis based on VADER (Hutto and Gilbert 2014) and the generic Employee Resilience Scale (Näswall et al. 2015), which we adapted to the remote work context, we first found that sentiment scores for the high STS alignment group are significantly higher than for the low STS alignment group. This indicates a significant difference in how people feel about remote work depending on their level of STS resilience. Second, resilience towards remote work is also significantly higher for the high STS alignment group when compared to the low STS alignment group. We thereby show that personal digital resilience relates positively to STS resilience. Finally, we found that sentiment scores when describing remote work situations are significantly related to personal resilience scores. This implies, that measurement tools aimed at capturing individual resilience levels in a specific context might benefit from incorporating sentiment scores. Overall, our results indicate that employees embedded in a resilient STS that is highly aligned to the new digital conditions of work also show higher personal digital resilience and communicate more positively about remote work. This implies that the measures proposed above are likely to not only improve the overall STS resilience but also the personal digital resilience of employees. However, as employee resilience is influenced by a variety of additional factors across individual, family organizational, community and national levels, there are many direct approaches to improving it beyond the scope of our paper, such as resilience training that fosters the building of competencies related to resilience and which will be of benefit when the next disruption hits (Robertson et al. 2015).

Another contribution refers to the broadening of our understanding of (successful) realignment processes by using the sociotechnical systems perspective. Our findings show that all components of STS need to be realigned to reach a new stable state (Holling 1973; Vogus and Sutcliffe 2007) in times of externally enforced changes towards integration of remote work (Kurland and Bailey 1999; Stein et al. 2015; Molino et al. 2020; Wang et al. 2020). In particular, the interaction of the social and technical components, which is the core of the STS framework (Sarker et al. 2019) is crucial. Beyond the alignment towards integrated remote work, our findings support the characterization of the STS framework by Sarker et al. (2019) as a core theoretical fundament for understanding information systems dynamics. We have shown that the sentiments expressed in individuals’ descriptions of sociotechnical systems mirror the degree of success in reaching a new stable state when realigning to a changed situation (Holling 1973; Vogus and Sutcliffe 2007). This might serve as a theoretical basis for further research in information systems resilience (Erol et al. 2010; Smith et al. 2011; Sarkar et al. 2017).

Our research offers several opportunities for future work. Firstly, it remains unclear whether the human or technical side of STS has a stronger influence on how successful it realigns overall. By investigating causality, we can discover whether personal digital resilience drives STS to become more resilient or the other way around. This will help organizations prioritize which parts of STS to target first with interventions to support its ability to realign. Causal effects can also be expected from the relations to sentiments because research shows that resilient people feel and communicate more positivity in times of crises while, on the other hand, the experience of positive emotions in turbulent times can prompt individuals towards gaining resilience (Tugade and Fredrickson 2004; Kohn 2020).

As our sample size is quite small and the participants are from one country and hold a limited range of working roles, our results may not be generalizable to employees from other countries, cultures, or in different working domains. Since Q methodology does not aim at a large sample size (Watts and Stenner 2005), future work could develop a different method to measure STS resilience and validate our results with a larger sample size. Due to our research design we could not measure pretreatment characteristics such as alignment levels of participants’ STS prior to the pandemic. Conducting longitudinal studies to overcome this limitation seems to be a promising research endeavor. Longitudinal studies, for instance, may analyze sociotechnical systems resilience development over time beyond the COVID-19 pandemic. Such research will help increase understanding of how to cultivate digital resilience (Zhang et al. 2022). Finally, scholars could study the impact of, for example, organizational commitment or job engagement (Perry et al. 2018) on the level of digital resilience.

Though we find control variables to have little impact on whether employees adjust well to IS supported changes or not, we highly recommend considering them for further investigations. For instance, we expect social isolation to be more of an issue in single households, as they have fewer possibilities to balance the lack of social interactions due to remote work (Kurland and Bailey 1999). Moreover, we cannot claim that we elicited all possible attitudes regarding employees’ experiences with remote work during COVID-19 (McKeown and Thomas 1988).

## Conclusion

Due to the COVID-19 pandemic, many organizations were forced to have their employees work from home, resulting in a realignment of processes, technology, and people. By applying Q methodology, this research paper shows that employees differ significantly with respect to their subjective views on remote work and the sociotechnical systems they work and live with. We find that higher levels of STS alignment cause higher sentiment scores associated with positivity when describing remote work situations. We also find that higher levels of STS alignment cause higher degrees of employee resilience in remote work settings. Our results have vast practical implications for realigning STS toward a new stable state. For instance, we confirm that the interdependence of both the technical and social components in sociotechnical systems is crucial for STS alignment processes. Harmony between both components results in better instrumental and humanistic outcomes. Experienced technical and social support, for instance, relates to the willingness and ability to learn new things when working remotely, high performance and close contact with colleagues, less technostress, and adherence to security guidelines.

## Supplementary Information

Below is the link to the electronic supplementary material.

Supplementary file1 (PDF 468 kb)

## Footnotes

1 www.deepl.com/translator.

2 It is unlikely that incorrect translations affect the sentiment scoring of the text. For instance, "work" has a female definite article in German and that's why a comment is translated to "She is okay, but in the long run it is no fun" where "she" refers to the word “work”. It would have been correct to refer to work as "it", which, however, does not change the sentence's sentiment in VADER.

3 The German adaptation of the lexicon and rule-based sentiment analysis tool VADER, known as GerVader, lacks recognition of negations, contrast conjunctions, and context information (Tymann et al. 2019).

4 We note that there is no consensus in the literature as to how many statements to include in a Q sample. Reliable results are reported based on as few as ten statements (Rogers 1995), while others suggest up to 140 statements (Kerlinger 1973).

5 The R methodology describes non-Q methodologies, such as test theory, and is characterized by a priori assumptions and results that are seldom operational (McKeown and Thomas 2013).

6 https://www.clickworker.com/about-us/. ClickWorker offers access to over two million registered independent “ClickWorkers,” with supervision and reputation mechanisms (similar to MTurk). ClickWorker also offers the ability to sample participants based on a variety of characteristics, such as age, gender, country, etc. Common tasks completed by ClickWorkers include completing surveys, assessing data quality and classifying and categorizing information, similar to the tasks commonly found on MTurk.
