# Supplementary material for: How Sociotechnical Realignment and Sentiments Concerning Remote Work are Related – Insights from the COVID-19 Pandemic
Source: Bus Inf Syst Eng. 2023 Mar 24:1–18. Online ahead of print. doi: 10.1007/s12599-023-00798-8 (PMC10037393; doi:10.1007/s12599-023-00798-8)
Supplement: Supplementary file 1 — Supplementary file1 (PDF 468 kb) [file 12599_2023_798_MOESM1_ESM.pdf]

How Sociotechnical Realignment and Sentiments Concerning Remote Work are Related – Insights from the COVID-19 Pandemic

Vanessa Kohn, Muriel Frank, Roland Holten

Business & Information Systems Engineering (2023)

Appendix (available online via http://link.springer.com)

Appendix

Appendix A: Complete list of all Q sort statements

#	Q sort statements (translated into English by a professional translator)	Potential aspects of STS alignment	Source	STS framework component
1	I talk to my colleagues via video or voice call on a daily basis.	OTS1	Sarkar et al. (Sarkar et al. 2017)	Social
2	I sometimes have informal calls with my colleagues to talk about nonwork-related topics.	OTS2	Sarkar et al. (Sarkar et al. 2017)	Social
3	Fulfilling my work tasks is highly interdependent with the work of others.	OTS3	Eckhardt et al. (Eckhardt et al. 2019)	Social
4	I still collaborate with my colleagues, but it feels more orchestrated and less spontaneous.	OTS4	Waizenegger et al. (Waizenegger et al. 2020)	Social
5	I am more likely to participate in informal Friday afternoon meetings because I don't need to worry about being late home from the office.	OTS5	Waizenegger et al. (Waizenegger et al. 2020)	Humanistic
6	I feel more connected to my colleagues than I ever did before.	OTS6	Waizenegger et al. (Waizenegger et al. 2020)	Social
7	Since working from home, I have more contact at work with people I normally interact less with.	OTS7	Waizenegger et al. (Waizenegger et al. 2020)	Social
8	My manager tracks my performance when working remotely.	OMS1	Eckhardt et al. (Eckhardt et al. 2019)	Instrumental
9	I feel like my managers do not provide any emotional support during the COVID-19 pandemic.	OMS2	Eckhardt et al. (Eckhardt et al. 2019)	Social
10	My managers helped me understand the differences in communication, technology, and self-discipline when transitioning to remote work.	OMS3	Eckhardt et al. (Eckhardt et al. 2019)	Social
11	I know exactly when and how to reach my managers when working remotely.	OMS4	Eckhardt et al. (Eckhardt et al. 2019)	Social
12	My manager continuously ensures me I can be productive at home.	OMS5	Wade and Shan (Wade and Shan 2020)	Social
13	My manager communicates precise and realistic objectives to me.	OMS6	Wade and Shan (Wade and Shan 2020)	Social

14	Management asked for my feedback on how to optimize my remote work experience.	OMS7	Sakurai and Chughtai (Sakurai and Chughtai 2020)	Social
15	I participated in workshops on data protection or IT security.	OTT1	Sarkar et al. (Sarkar et al. 2017)	Joint
16	My organization offered training to prepare for the new network structures when working remotely.	OTT2	Eckhardt et al. (Eckhardt et al. 2019)	Joint
17	My organization offers tool training.	OTT3	Eckhardt et al. (Eckhardt et al. 2019)	Joint
18	My company provided me with the necessary equipment to be able to work from home.	OTT4	Scheppers and Middleton (Scheepers and Middleton 2013)	Technical
19	Working from home makes it hard to comply with my organization's information security policies.	OTT5	Li and Siponen (Li and Siponen 2011)	Joint
20	Making use of workarounds with respect to security rules is fine during a pandemic.	OTT6	Anderson and Agarwal (Anderson and Agarwal 2010)	Joint
21	I care a lot about protecting my data.	ISC1	Sarkar et al. (Sarkar et al. 2017)	Joint
22	I am worried about security risks when working from home.	ISC2	Sarkar et al. (Sarkar et al. 2017)	Joint
23	I can get my tasks done much faster when working from home.	IPP1	Waizenegger et al. (Waizenegger et al. 2020)	Instrumental
24	I feel like remote work is a barrier to learning new things at work.	IPP2	Waizenegger et al. (Waizenegger et al. 2020)	Instrumental
25	It is challenging for me to manage non-work-related sources of stress while working from home.	ITS1	Eckhardt et al. (Eckhardt et al. 2019)	Humanistic
26	I experience more technostress since working from home.	ITS2	Eckhardt et al. (Eckhardt et al. 2019)	Humanistic
27	When something doesn't go right at work, I take out my anger on the people living with me.	IWH1	Benlian (Benlian 2020)	Humanistic
28	When I successfully complete a task at work, I share my good mood with the people living with me.	IWH2	Benlian (Benlian 2020)	Humanistic
29	Unscheduled work calls invade my privacy at home.	IWH3	Waizenegger et al. (Waizenegger et al. 2020)	Humanistic

30	I still don't know a lot of functions of the tools I use to work from home.	ISE1	Sarker et al. (Sarkar et al. 2017)	Technical
31	I could complete my job using a new software package if I had never used one like this before.	ISE2	Wang and Haggerty (Wang and Haggerty 2011)	Technical
32	Sometimes I set my status to unavailable when I don't want to be reached.	IDW1	Eckhardt et al. (Eckhardt et al. 2019)	Humanistic
33	I often feel bad when I don't answer messages immediately.	IDW2	Eckhardt et al. (Eckhardt et al. 2019)	Humanistic
34	It is important for me to be up to date about technological trends.	IPB1	Sarkar et al. (Sarkar et al. 2017)	Humanistic
35	I don't actively search for news on technological developments.	IPB2	Sarkar et al. (Sarkar et al. 2017)	Humanistic
36	I don't feel comfortable turning on my camera in large online meetings.	IPB3	Sarkar et al. (Sarkar et al. 2017)	Humanistic
37	The idea of living in a digitalized world is repulsive to me.	IPB4	Sarkar et al. (Sarkar et al. 2017)	Humanistic
38	I immediately understood the need to shift to remote work during the pandemic.	IPB5	Sarkar et al. (Sarkar et al. 2017)	Humanistic
39	Without separate interests, I think it's harder to make use of the advantages that come with full-time remote work.	IPB6	Eckhardt et al.(Eckhardt et al. 2019)	Humanistic
40	I believe remote work plays an important role in a modern society.	IPB7	Raghuram et al. (Raghuram et al. 2010)	Humanistic

OTS = Team support
OMS = Management support
OTT = Technical support / training
ISC = Security concerns
IPP = Perceived performance
ITS = Technostress
IWH = Work-home conflict
ISE = Self-efficacy
IDW = Digital well-being
IPB = Personal belief / preference

Additional References in Appendix A

Anderson CL, Agarwal R (2010) Practicing safe computing: a multimethod empirical examination of home computer user security behavioral intentions. MIS Q 34:613–643. https://doi.org/10.2307/25750694

Li Y, Siponen M (2011) A call for research on home users' information security behaviour. In: Proceedings of the 16th Pacific Asia Conference on Information Systems. Brisbane

Raghuram S, Tuertscher P, Garud R (2010) Mapping the field of virtual work: a co-citation analysis. Inf Syst Res 21:983–999. https://doi.org/10.1287/isre.1050.0050

Scheepers R, Middleton C (2013) Personal ICT ensembles and ubiquitous information systems environments: key issues and research implications. Commun Assoc Inf Syst 33:381–392. https://doi.org/10.17705/1CAIS.03322

Appendix B: Modifications to automatic sentiment score

Participant's comments on their remote work experience	VADER score	Modified score	Explanation for modification
Everything has its advantages and disadvantages. It is important to have a job at all.	**0.1531**	**0.0**	**(2)**
More mixed. I see clear advantages: Saving time, more equal meetings, better compatibility of professional and private concerns. What I miss most of all are the personal meetings and contacts. Creative work works worse than face-to-face contact.	**0.7553**	**0.0**	**(2)**
I still find it much more productive than in the office, where one is constantly "harassed".	**-0.5423**	**0.3**	**(1)**
A good thing, if you only had the equipment (own study, sufficiently fast Internet).	**0.4404**	**0.0**	**(2)**
In certain phases this can be quite useful, but in the long run, office work cannot be adequately replaced by the home office.	**0.3912**	**0.0**	**(2)**
I actually don't care where I work from. But it would be nice to have the choice. This is not given with a current office capacity of 25%. Here, I leave the priority to colleagues who do not have the infrastructure at home, e.g. a separate study.	**0.379**	**0.0**	**(2)**
Working from home benefits many people, for example if you have appointments or children, because it allows you to work more flexibly. But I think that after the pandemic more people should work in the office again, because the group feeling is stronger and that is important.	**0.799**	**0.0**	**(2)**
Even though the home office has many advantages and I can work more efficiently, I lack the social and personal interaction with colleagues. Especially because I live alone, I quickly become lonely.	**-0.2458**	**0.0**	**(2)**
In Corona times, home office is a sensible and necessary measure, but if I could choose it, I would usually want to work in the office.	**0.1154**	**0.0**	**(2)**
Very good, because I save an hour of commuting time. At the same time, I was afraid of being more closely monitored. I was unsure whether I would not be less productive. And I wondered how I should work with the equipment I have.	**0.659**	**0.0**	**(2)**
couldn't imagine that the established "paper processes" would also work digitally\- but: I was able to reconcile family and career better immediately.	**0.5927**	**0.0**	**(2)**
On the one hand, I felt a little insecure because of the change and at first I felt overwhelmed with the new tasks and procedures, on the other hand, I felt relieved and more confident that I could stay at home more because of the health situation.	**0.8803**	**0.0**	**(2)**
I was sympathetic because at the beginning of the pandemic I was very worried about becoming infected with SARS-COV 2 and wanted to isolate myself.	**-0.4927**	**0.3**	**(1)**

The situation was initially very unfamiliar to me, as working from home did not play a role in my company before.	**0.2584**	**0.0**	**(2)**
Very unsafe!	**0.0**	**-0.3**	**(3)**
It's an unusual feeling, since I only started working after Corona had been in progress for a while, which meant that I didn't get to know many colleagues personally. This makes it very difficult to integrate into a team.	**0.1298**	**-0.3**	**(3)**
It wasn't a big emotional change for me, since I had already done most of my work from home before Corona.	**-0.1139**	**0.0**	**(2)**
I was happy that I now have more freedom to organize my daily work routine but was also afraid of social isolation, especially because I live alone.	**0.2391**	**0.0**	**(2)**
safer, more isolated, detached from the team, but as a risk patient had no alternative.	**-0.6942**	**0.0**	**(2)**

Explanation Index

(1) The comment contains only positive feelings toward remote work. The algorithm did not understand the context or negotiation of negative words correctly. We manually changed the score to 0.3 to indicate a positive sentiment (Domagalski 2020).

(2) The comment either contains a well-balanced mix of positive and negative sides of remote work leading to a neutral overall score or only states a neutral fact such as familiarity with remote work prior to the pandemic.

(3) The comment contains only negative feelings toward remote work. We manually changed the score to -0.3 to indicate a negative sentiment (Domagalski 2020).

Appendix C: Testing control variables

**Fisher's Exact Test**	
Age	p-value = 0.8387
Gender	p-value = 0.2953
Education	p-value = 0.9352
Marital status	p-value = 0.0438
Living situation	p-value = 0.1492
Type of employment	p-value = 1.0000
Month	p-value = 0.4817

Contingency Table of Marital Status by Groups

	**Single**	**Relationship or married**	**Divorced or widowed**	**Total**
High STS alignment group	8	17	1	26
Low STS alignment group	9	3	0	12
Total	17	20	1	38

Appendix D: Testing assumptions of linear regression for (Sentiments ~ Factor Loadings)

(Sentiments ~ Loadings on Factor 1)

**Durbin-Watson-Test**			
**Lag**	**Autocorrelation**	**D-W Statistic**	**p-value**
1	-0.1509978	2.28181	0.386

**Breusch-Pagan test**		
**BP**	**df**	**p-value**
1.194	**1**	0.2745

**Collinearity Statistics**	
	**VIF**
Loadings1	1

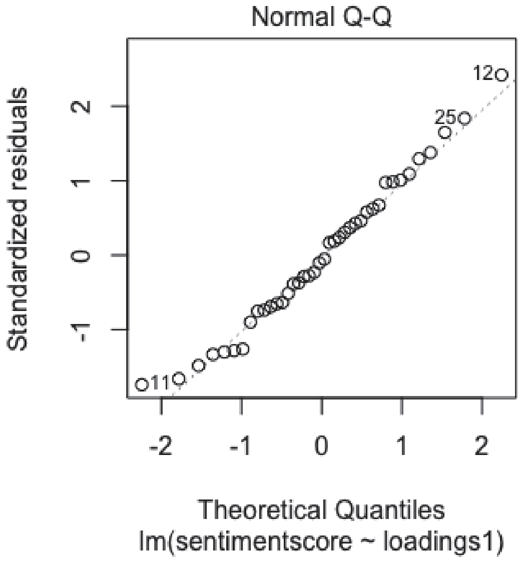

(Sentiments ~ Loadings on Factor 2)

**Durbin-Watson-Test**			
**Lag**	**Autocorrelation**	**D-W Statistic**	**p-value**
1	-0.1649922	2.325687	0.296

Breusch-Pagan test		
**BP**	**df**	**p-value**
0.26924	1	0.6038

Collinearity Statistics	
	**VIF**
Loadings2	1

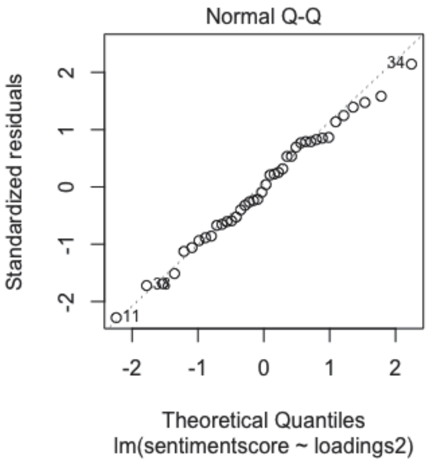

Appendix E: Testing assumptions of linear regression for (Personal Digital Resilience ~ Loadings on Factor 2)

(Personal Digital Resilience ~ Loadings on Factor 1)

**Durbin-Watson-Test**			
**Lag**	**Autocorrelation**	**D-W Statistic**	**p-value**
1	0.04660695	1.894073	0.684

**Breusch-Pagan test**		
**BP**	**df**	**p-value**
0.4013	1	0.5264

**Collinearity Statistics**	
	**VIF**
Loadings1	1

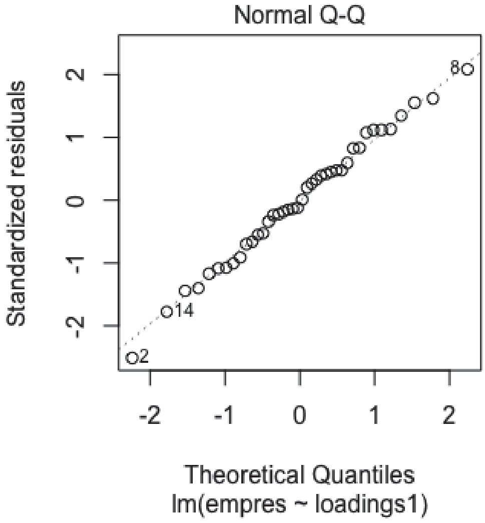

(Personal Digital Resilience ~ Loadings on Factor 2)

**Durbin-Watson-Test**			
**Lag**	**Autocorrelation**	**D-W Statistic**	**p-value**
1	0.02589249	1.88883	0.72

Breusch-Pagan test		
**BP**	**df**	**p-value**
0.038928	1	0.8436

Collinearity Statistics	
	**VIF**
Loadings2	1

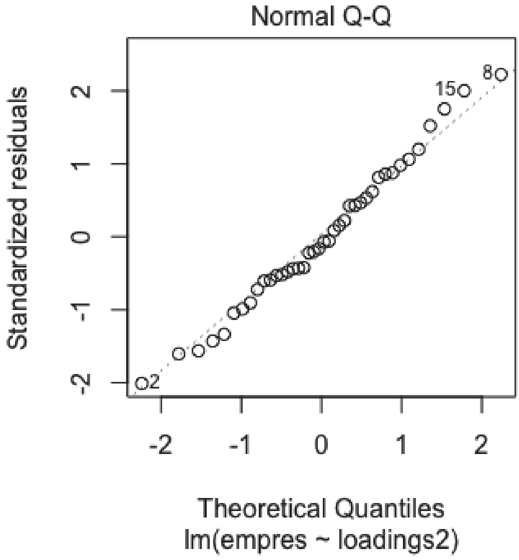

Appendix F: Testing assumptions of linear regression for (Sentiments ~ Resilience)

Durbin-Watson-Test			
**Lag**	**Autocorrelation**	**D-W Statistic**	**p-value**
1	-0.1095363	2.174439	0.52

Breusch-Pagan test		
**BP**	**df**	**p-value**
0.16822	1	0.6817

Collinearity Statistics	
	**VIF**
Resilience	1

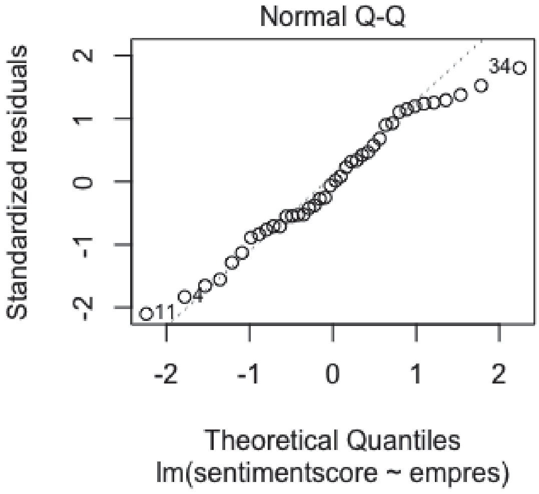
Normal Q-Q
Standardized residuals
Theoretical Quantiles
lm(sentimentscore ~ empres)
34
11
4
